# Development of Machine Learning Model for VO_2max_ Estimation Using a Patch-Type Single-Lead ECG Monitoring Device in Lung Resection Candidates

**DOI:** 10.3390/healthcare11212863

**Published:** 2023-10-30

**Authors:** Hyun Ah Lee, Woosik Yu, Jong Doo Choi, Young-sin Lee, Ji Won Park, Yun Jung Jung, Seung Soo Sheen, Junho Jung, Seokjin Haam, Sang Hun Kim, Ji Eun Park

**Affiliations:** 1 Department of Pulmonary and Critical Care Medicine, Ajou University School of Medicine, Suwon 16499, Republic of Korea; 2 Department of Thoracic and Cardiovascular Surgery, Ajou University School of Medicine, Suwon 16499, Republic of Korea; yws081011@aumc.ac.kr (W.Y.);; 3 Seers Technology Co., Seongnam-si 13558, Republic of Korea; 4 Department of Rehabilitation Medicine, Biomedical Research Institute, Pusan National University Hospital, Busan 49241, Republic of Korea

**Keywords:** maximal oxygen consumption (VO_2max_), cardiopulmonary exercise test (CPET), machine learning model, estimation, lung resection candidates

## Abstract

A cardiopulmonary exercise test (CPET) is essential for lung resection. However, performing a CPET can be challenging. This study aimed to develop a machine learning model to estimate maximal oxygen consumption (VO_2max_) using data collected through a patch-type single-lead electrocardiogram (ECG) monitoring device in candidates for lung resection. This prospective, single-center study included 42 patients who underwent a CPET at a tertiary teaching hospital from October 2021 to July 2022. During the CPET, a single-lead ECG monitoring device was applied to all patients, and the results obtained from the machine-learning algorithm using the information extracted from the ECG patch were compared with the CPET results. According to the Bland–Altman plot of measured and estimated VO_2max_, the VO_2max_ values obtained from the machine learning model and the FRIEND equation showed lower differences from the reference value (bias: −0.33 mL·kg^−1^·min^−1^, bias: 0.30 mL·kg^−1^·min^−1^, respectively). In subgroup analysis, the developed model demonstrated greater consistency when applied to different maximal stage levels and sexes. In conclusion, our model provides a closer estimation of VO_2max_ values measured using a CPET than existing equations. This model may be a promising tool for estimating VO_2max_ and assessing cardiopulmonary reserve in lung resection candidates when a CPET is not feasible.

## 1. Introduction

Lung cancer is the second most commonly diagnosed cancer and the leading cause of cancer-related mortality [1]. Surgical resection is considered the best curative option for lung cancer [2]. However, lung cancer patients are often elderly, have weakened lung function due to smoking, or have underlying medical conditions [3,4]. Although surgery may be feasible based on the cancer stage, some patients opt for alternative treatments due to concerns about post-surgical complications. Fortunately, advancements in surgical techniques and tools have decreased morbidity and mortality rates, and more patients are now eligible for lung resection [5,6].

Before lung resection, the patient’s risk and ability to tolerate the procedure were thoroughly evaluated. This evaluation includes several tests, such as the pulmonary function test, 6-min walking test, and cardiopulmonary exercise test (CPET) [7]. In particular, the CPET helps assess patients’ cardiopulmonary reserve by measuring cardiac output, ventilation, oxygen uptake, and carbon dioxide output during exercise. Therefore, several guidelines recommend it as an important predictor of post-thoracotomy morbidity and mortality in high-stress situations, such as lung surgery and the immediate postoperative period. According to the British Thoracic Society (BTS), American College of Chest Physicians (ACCP), and European Respiratory Society (ERS) guidelines, some patients who are not considered suitable for surgery based on forced expiratory volume in one second (FEV_1_) and forced vital capacity (FVC), or diffusing capacity of the lung for carbon monoxide (DLco) may still be able to undergo the procedure if the results of the CPET permit it [8,9,10].

The CPET is a reliable method that ensures precise and reproducible results by continuously monitoring multiple parameters in a controlled environment. In particular, guidelines provide standards for measuring oxygen consumption (VO_2_), such as maximal oxygen consumption (VO_2max_) [9] and peak VO_2_ [8,10]. However, owing to the high cost of equipment such as gas analyzers and the need for trained technicians to collect and interpret data, the use of VO_2_ assessments for risk prediction in clinical practice is limited [11]. To overcome these limitations in utilizing the CPET in clinical settings, two organizations, the American College of Sports Medicine (ACSM) and the Fitness Registry and Importance of Exercise National Database (FRIEND), have developed equations that estimate VO_2max_ using information about patients and their exercise tests [12,13,14]. These equations are frequently employed in evaluating physical activity in patients and clinical studies [15,16].

Recently, with the development of various wearable devices, the evaluation and tracking of an individual’s physical activity have become more accessible. These devices provide valuable information that helps identify a patient’s baseline condition, including daily steps, heart rate, energy expenditure, and cardiorespiratory fitness. An electrocardiogram (ECG) patch is an example of wearable technology used in remote monitoring equipment to track patients’ regular activities or rehabilitation exercises at home and send clinical data to medical staff. Wearable devices play a crucial role in monitoring due to their potential capabilities [17].

This study aimed to develop a machine learning model to estimate VO_2max_ using data collected through a patch-type single-lead ECG monitoring device during maximal exercise tests in patients with pulmonary disease who are candidates for lung resection. Furthermore, the VO_2max_ estimated through the machine learning model and the VO_2max_ obtained through the clinical equations, which are the previously used estimated VO_2max_ equations, were compared with the actual VO_2max_ measured through the CPET. Through this study, we sought to evaluate the usefulness of a machine learning model in estimating VO_2max_ for patients requiring lung resection surgery with limited exercise capacity or when a CPET is not feasible due to resource constraints.

## 2. Materials and Methods

### 2.1. Study Design and Subjects

This prospective single-center study was performed at a tertiary teaching hospital in South Korea between October 2021 and July 2022. Eligible participants were adults (aged ≥19 years) who had been diagnosed with cancer through biopsy or were strongly suspected of having cancer without tissue examination results, leading to scheduled lung resection. Only patients who voluntarily agreed to participate in the study were enrolled. Patients with contraindications to a CPET were excluded, including resting O_2_ saturation levels < 85%, acute coronary insufficiency, uncontrolled arrhythmia, decompensated heart failure, and acute infection [18]. Patients who had previously experienced allergic reactions to the investigational devices or their components were also excluded.

As part of the preoperative evaluation, all patients underwent a CPET. Before starting the CPET, a patch-type single-lead ECG monitoring device (modiCARE-MC100; SEERS Technology, Pyeongtaek-si, Gyeonggi-do, Republic of Korea) was attached to the skin in front of the chest for examination. This wearable device was kept in place until the completion of the CPET. Therefore, all patients were examined by simultaneously attaching the patch-type single-lead ECG monitoring device during the CPET, and the results obtained from the ECG patch were compared with the CPET results, which are considered the gold standard test.

### 2.2. Cardio-Pulmonary Exercise Test (CPET)

The patients underwent exercise stress testing on a treadmill using a modified Bruce protocol. The treadmill speed and slope were changed every 3 min according to the protocol (Table S1). The exercise test was terminated according to the indications provided by the American Heart Association (AHA) guidelines, such as ischemic ECG changes, sustained ventricular tachycardia, a drop in systolic blood pressure >10 mmHg accompanied by any other evidence of ischemia, moderate-to-severe angina, central nervous system symptoms (e.g., dizziness, near syncope), signs of poor perfusion (cyanosis or pallor), and the subject’s request to stop [19].

A respiratory gas analyzer (Quark PFT; COSMED Co., Rome, Italy), automatic blood pressure and pulse monitor (TANGO M2; SunTech Medical Inc., Morrisville, NC, USA), and treadmill with an ECG monitoring system (T2100-ST2; GE-Marquette Medical Systems, Milwaukee, MI, USA) were used during the CPET.

### 2.3. Measuring Device (modiCARE-MC100, MC-100)

The MC-100 had two circular electrodes 120 mm apart. It can record a single-lead ECG with a sampling rate of 256 Hz and send real-time ECG recording data to the research smartphone application through Bluetooth. The electrodes were placed at a 45-degree angle from the internipple line, which is considered the optimal location for single-lead ECG monitoring devices [20].

This device also includes a three-axis accelerometer and three-axis gyroscope sensors, which record at a sampling rate of 50 Hz and are utilized to calculate movement and respiration.

### 2.4. Developing a Machine Learning Algorithm to Predict VO_2max_

#### 2.4.1. Measurement Variables by MC-100: Heart Rate, Acceleration, and Gyroscope

The MC-100 recorded the heart rate for each heartbeat and measured the acceleration (ACC) and gyroscope (Gyro) at a rate of 50 data points per second. However, the CPET, the reference device, only stores respiratory gas and ECG data once every 15 s. Consequently, we calculated the average heart rate, ACC, and Gyro data over 15 s to extract values at the same intervals as the CPET device.

**Figure 1:**
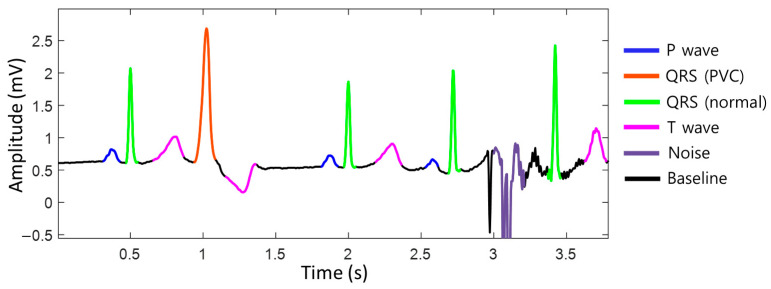
The segmentation of the electrocardiogram (ECG). The ECG data are standardized into baseline, P-wave, normal QRS complex, premature ventricular contraction (PVC) QRS complex, T-wave, noise, and fibrillation probabilities per sample. ECG, electrocardiogram; QRS complex, a combination of the Q, R, and S waves; PVC, premature ventricular contraction.

To achieve accurate heart rate monitoring during exercise, unstructured raw ECG data were standardized into a baseline, p-wave, normal QRS complex, premature ventricular contraction (PVC) QRS complex, T-wave, noise, and fibrillation probabilities on a per-sample basis using deep learning semantic segmentation algorithms (Figure 1). By distinguishing between normal heartbeats and noise, it is possible to accurately assess whether the situation is noise-related, allowing for the measurement of the heart rate that closely approximates the actual heart rate, even during exercise. We utilized the DDRNet-23-slim (Deep Dual-Resolution Networks) architecture, which is a type of semantic segmentation algorithm [21]. This approach allowed us to accurately differentiate between different ECG waveforms using high- and low-resolution feature maps, which are key components of the DDRNet-23-slim model.

The input of this model is set to a 16-s window to capture both local and global features of the ECG. The model parameters are optimized to reflect ECG characteristics. All the data used for training were labeled by medical experts, indicating the start and end points of the baseline, p-wave, normal QRS complex, premature ventricular contraction (PVC) QRS complex, T-wave, noise, and fibrillation (Figure S1).

#### 2.4.2. Feature Selection

We extracted all possible data that could be utilized to develop machine learning models to estimate VO_2max_ and developed a regression model. First, the three items measured by the MC-100 (heart rate, ACC, and Gyro) were checked for correlation with VO_2_. We found that all three measurements increased as exercise intensity increased and had a correlation coefficient of ≥0.7 (Figure S2). In addition to these, three basic items of information–age, height, and body weight–were also included as variables.

Second, when confirming the correlation between the six variables, the ACC and Gyro showed a high correlation (Figure S3). To avoid multicollinearity, Gyro signals were excluded from the input variables. The removal of highly correlated features enhances the reliability and stability of the model while minimizing the risk of overfitting. As part of the feature selection, we conducted an ablation test to assess the performance of our model by gradually removing the features from the five remaining items. During this process, we obtained regression model evaluation metrics for each combination: R-squared (R^2^), mean absolute error (MAE), mean squared error (MSE), and root mean square error (RMSE) (Table 1). According to the results, we determined that heart rate, ACC, and body weight were the most practical combinations of features. Finally, we developed regression models that utilized only these three features.

#### 2.4.3. Model Development

We compared seven regression models to evaluate their estimation performance: linear, quadratic, cubic, ridge, lasso, elastic, and random forest. To evaluate and compare the performance of the models, we considered four commonly used metrics in time-series analysis. These metrics included R^2^ score, MAE, MSE, and RMSE. We found that quadratic regression performed the best; therefore, it was used for model development (Table 2).

We extracted heart rate, ACC, and Gyro data from the MC-100 every 15 s to align with our reference device, the CPET equipment. As a result, the total number of data is 1249, and the 40 participants on average have 31 data per person. Data were randomly split into training and test data in a 4:1 ratio, and the models were internally validated using the test data. The machine-learning models were trained with fivefold cross-validation during the training process, and the test data were used to measure the model performance.

#### 2.4.4. Comparison between the Machine-Learning Model and Clinical Equation

To evaluate the estimation performance of the developed model, it was compared with equations from the American College of Sports Medicine (ACSM), Fitness Registry and Importance of Exercise National Database (FRIEND), and FRIEND equation for patients with heart failure (HF-FRIEND), which are existing formulas for estimating VO_2max_. These equations have been previously developed and are widely used. The three formulas are as follows:

- ACSM equation
  VO_2max_ = [speed (m/min) × (0.1 + fractional grade × 1.8) + 3.5];
- FRIEND equation
  VO_2max_ = [speed (m/min) × (0.17 + fractional grade × 0.79) + 3.5];
- HF-FRIEND equation
  VO_2max_ = [speed (m/min) × (0.17 + fractional grade × 0.32) + 3.5].
- The “speed” and “fractional grade” represent the values corresponding to the stage at which the patient achieved their maximum during a CPET conducted according to the modified Bruce protocol.

### 2.5. Statistical Analysis

The baseline characteristics of the cohort were analyzed using descriptive statistics. Continuous variables were expressed as mean ± standard deviation, while categorical variables were expressed as frequencies (percentages). Statistical significance was determined using two-sided *p*-values of <0.05, with 95% confidence intervals excluding 0.

The Bland–Altman plot is an analysis based on quantifying the agreement between two quantitative measurements by indicating the mean difference and constructing limits of agreement. The resulting graph is an XY scatter plot, in which the *Y*-axis shows the difference between the two paired measurements, and the *X*-axis represents the mean of these measures. In other words, the difference between two paired measurements was plotted against the mean of the two measurements. The plots also indicate the mean differences between two paired measurements and the upper and lower limits of agreement. The limits of agreement were calculated as means ± 1.96 × standard deviation (SD). If the difference between the two methods was within the limits of agreement, it was judged appropriate [22].

The intraclass correlation coefficient (ICC) measures the agreement between the gold standard and tested devices. This provided an estimate of the overall concordance between the two methods. ICC values of ≥0.90 are considered excellent, values of 0.75–0.90 are good, values of 0.60–0.75 are moderate, and values of ≤0.60 indicate low agreement [23].

Descriptive statistics and ICC were analyzed using IBM SPSS Statistics for Windows, Version 29.0. (Armonk, NY, USA: IBM Corp; 2022). Statistical analyses, including classification, clustering, regression, model selection, and preprocessing, were performed using Python 3.6.13 (Reference Manual, Scotts Valley, CA, USA: CreateSpace; 2009) with the sci-kit-learn library (0.24.2).

## 3. Results

### 3.1. Baseline Characteristics

**Figure 2:**
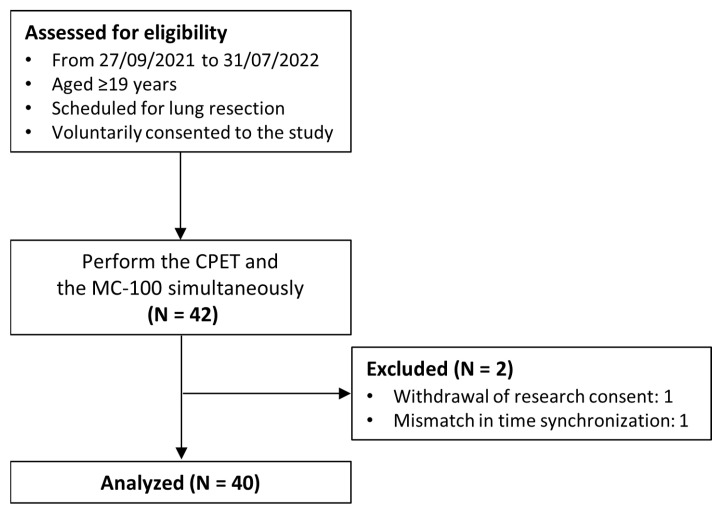
Flow diagram of the study.

A total of 42 patients were enrolled in the study; however, one participant withdrew from the study midway, and another participant had data synchronization issues that made the analysis impossible. Finally, 40 participants were included in the analysis (Figure 2), of whom 24 were males and 16 were females, with an average age of 66.7 years old (Table 3).

Among them, 21 (52.5%) had never smoked, and 19 (47.5%) had a smoking history. Ten patients had chronic obstructive lung disease and six patients had coronary artery disease. A total of 37 patients (92.5%) had Eastern Cooperative Oncology Group performance status scores of 0.

### 3.2. Pulmonary Function Test (PFT) and CPET

The mean FVC (% predicted), FEV_1_ (% predicted), and DL_CO_ (% predicted) were 90.7, 88.0%, and 77.7, respectively.

On the CPET, more than half (60%) of the patients reached stage 3, while only two patients advanced to stage 4. The observed maximal heart rate ratio was 87.4% of the predicted maximal heart rate value, and the measured VO_2max_ value was 26.3 ± 5.0 mL·kg^−1^·min^−1^.

### 3.3. Heart Rate Accuracy during Graded Exercise Testing

**Figure 3:**
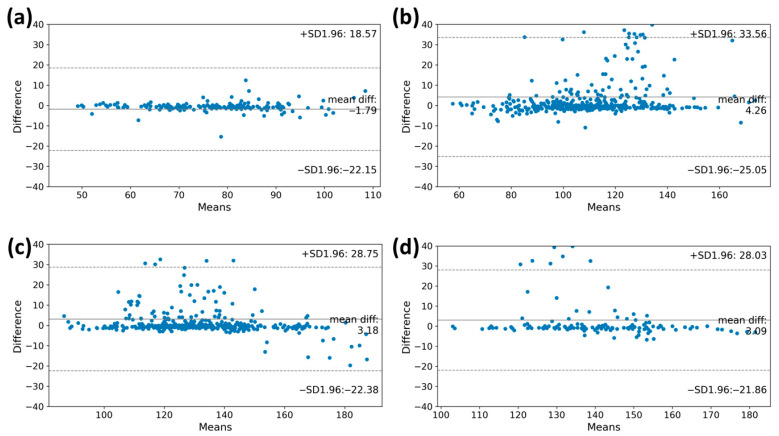
The Bland–Altman plots of the heart rate measurements at each stage of the CPET. These plots showed the degree of agreement between the two methods during graded exercise testing. The *y*-axis shows the differences in heart rate values of the CPET and MC-100, while the *x*-axis represents the mean heart rate values. The plots also indicate the mean differences between the estimated heart rate and heart rate from MC-100 and the upper and lower limits of agreement. (**a**) Warming up, (**b**) Stage 1, (**c**) Stage 2, and (**d**) Stage 3. CPET, cardiopulmonary exercise test.

For the high performance of the VO_2max_ estimation model, accurately measuring the heart rate through the MC-100 is crucial. We assessed the degree of agreement of the heart rates obtained using the MC-100 by comparing them with those obtained during the CPET. The Bland–Altman plots in Figure 3 illustrate the degree of agreement between the two methods during the graded exercise testing. A lower difference between the two measurements was observed in the warming-up stage (bias: −1.79 beats/min; limits of agreement: −22.15 beats/min to 18.57 beats/min). As the level goes up, the heart rate value measured through the MC-100 tends to be overestimated as the heart rate increases beyond 100 beats/min (bias: 4.26 beats/min; limits of agreement: −25.05 beats/min to 33.56 beats/min). In stage 2, when the heart rate exceeds 150 beats/min, the MC-100 tends to underestimate the heart rate (bias: 3.18 beats/min; limits of agreement: −22.38 beats/min to 28.75 beats/min). Despite these tendencies, it is important to note that most participants had heart rates within the consensus limits for both monitoring methods.

### 3.4. VO_2max_ Estimation

**Figure 4:**
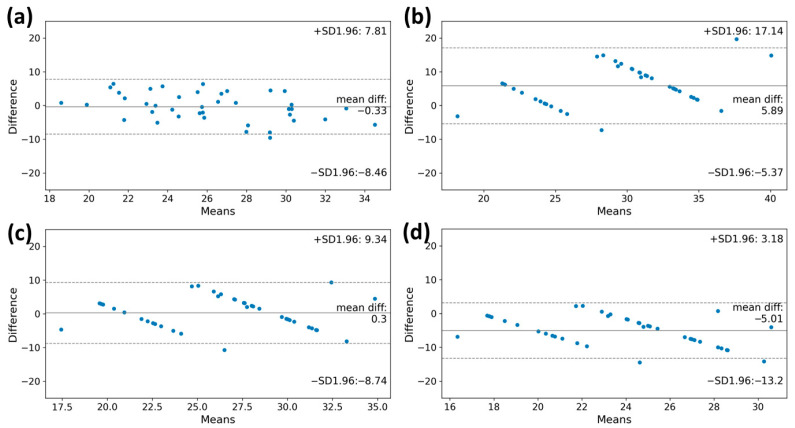
The Bland–Altman plots of the VO_2max_ estimation by estimation model and clinical equations. (**a**) machine learning model, (**b**) ACSM equation, (**c**) FRIEND equation, and (**d**) HF-FRIEND equation. The deviation between the estimated VO_2max_ value and the true measured value was minimal when using the machine learning model for estimation. VO_2max_, maximal oxygen consumption.

According to the Bland–Altman plot of measured and estimated VO_2max_, the VO_2max_ values obtained from the machine learning model and the FRIEND equation showed lower differences from the reference value (bias: −0.33 mL·kg^−1^·min^−1^; limits of agreement: −8.46 mL·kg^−1^·min^−1^ to 7.81 mL·kg^−1^·min^−1^, bias: 0.30 mL·kg^−1^·min^−1^; limits of agreement: −8.74 mL·kg^−1^·min^−1^ to 9.34 mL·kg^−1^·min^−1^, respectively). The VO_2max_ value calculated using the ACSM equation was overestimated compared to the reference (bias: 5.89 mL·kg^−1^·min^−1^; limits of agreement, −5.37 mL·kg^−1^·min^−1^ to 17.14 mL·kg^−1^·min^−1^) (Figure 4). Conversely, the VO_2max_ value derived from the HF-FRIEND equation was found to be underestimated (bias: −5.01 mL·kg^−1^·min^−1^; limits of agreement: −13.2 mL·kg^−1^·min^−1^ to 3.18 mL·kg^−1^·min^−1^).

Similarly, the machine learning model and the FRIEND equation showed a relatively high intraclass correlation coefficient (ICC) for VO_2max_ estimation, with values of 0.693 (95% CI, 0.417–0.838) and 0.708 (95% CI, 0.445–0.846), respectively (Table 4).

Subgroup analysis was performed according to the maximal stage reached by the patient during the CPET and according to sex. For analysis based on the maximum stage the patient reached, the patients were divided, with one group reaching stages 1 or 2 and the other group reaching stages 3 or 4. The machine learning model’s estimated VO_2max_ value showed mean differences of 0.32 mL·kg^−1^·min^−1^ and −0.68 mL·kg^−1^·min^−1^_,_ respectively, which were more accurate than other equations used for approximating actual VO_2max_ values. In subgroup analysis according to sex, the VO_2max_ values obtained from the machine learning model and the FRIEND equation showed lower differences from the reference value. For further details, please refer to Table 4 and Figure 4.

## 4. Discussion

This study aimed to develop a machine learning algorithm that can estimate VO_2max_ using a wearable device, a single-lead ECG patch (MC-100), in candidates for lung resection. To assess the level of agreement, we obtained the Bland–Altman plot and intraclass correlation coefficient (ICC) of our machine learning model and three other existing formulas. The results suggest that our model and the FRIEND equation can estimate VO_2max_ more closely than other formulas.

Because lung resection remains the best curative option for lung cancer, a preoperative evaluation is important. A CPET is frequently used to evaluate operability, especially in cases where PFT results are inoperable. Recently, the advancements in wearable technology and the growing attention to measuring physical activity have led to attempts to measure VO_2_, which requires specialized equipment. Several studies have reported favorable performance in measuring VO_2max_ and VO_2peak_ using a variety of wearable devices, including earbud-based sensors and watches, along with various protocols [24,25,26]. However, as they have predominantly targeted young and healthy adults, they have limitations in terms of their relevance for patient populations that require an evaluation of operability.

More recently, other studies have attempted to measure the VO_2max_ in patients rather than in healthy individuals. Greco et al. studied 31 elderly high-risk surgical patients using the Fitbit Inspire 2 to measure daily steps and VO_2max_, showing significant correlations (R = 0.56, *p* = 0.001 and r = 0.58, *p* = 0.006, respectively) with the actual 6-min walk test results [27]. In a study targeting patients scheduled for major elective intra-abdominal surgery, researchers demonstrated a significant correlation between physical activity levels measured over 7 days using the Garmin Vivosmart HR+ device and actual CPET parameters such as VO_2peak_ [28]. However, no studies have been conducted on patients scheduled for lung resection.

**Figure 5:**
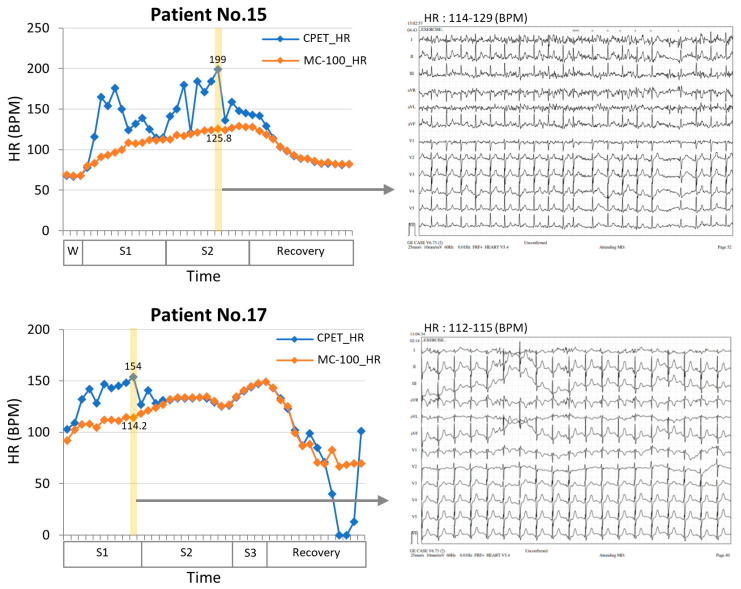
Comparisons of heart rate measurement between an electrocardiogram (ECG) and a single-lead ECG monitoring device during a CPET test in two patients. The results showed that the single-lead ECG monitoring device (MC-100) demonstrated significantly less fluctuation than the conventional ECG. In addition, when examining the 12-lead ECG (yellow section), it was found that the HR values had substantial discrepancies. However, the single ECG patch showed more approximate HR measurements and performed better in situations with high noise levels. W, warming up stage; S1, stage1; S2, stage2; S3, stage3; HR, heart rate; CPET, cardiopulmonary exercise test.

In this study, we developed a machine learning algorithm that can estimate VO_2max_ by utilizing wearable ECG monitoring equipment, MC-100, attached to lung resection candidates during exercise. The MC-100 device accurately measures the heart rate by minimizing the HR noise using its own algorithm. The Bland–Altman plots in Figure 3 show that the heart rates measured by the MC-100 device tended to be lower than those measured by the CPET equipment. However, upon reviewing the actual ECG data, it is clear that the measurements from the MC-100 device are more approximate to the real heart rate (Figure 5). In addition to the closer heart rate, by incorporating acceleration and weight data into the model, it was possible to estimate VO_2max_, which closely approximated the actual measured values.

VO_2max_ is a reliable indicator of aerobic capacity and cardiorespiratory function. Low levels of cardiorespiratory fitness in cancer patients have been associated with high symptom burden, morbidity, and mortality. However, the limitations of the CPET, which requires specialized equipment and skilled personnel, have restricted its use in clinical practice. Researchers have developed a formula for estimating VO_2max_ using exercise test characteristics. Our study utilized a machine learning model based on information collected using a single-lead ECG monitoring device to estimate VO_2max_. The model showed a moderate level of agreement, as indicated by an ICC value of 0.693. Compared to other equations, this model demonstrated greater consistency when applied to different maximal stage levels and sexes. When subgroup analysis was performed based on the maximal stage reached by the patients, these strengths became even more apparent. The machine learning model using the ECG monitoring device obtained personalized VO_2max_ values, while the equation-derived values remained the same for all patients who reached the same maximal stage. Therefore, when a CPET is not feasible, our model offers a potential solution for achieving higher accuracy in estimating VO_2max_.

**Figure 6:**
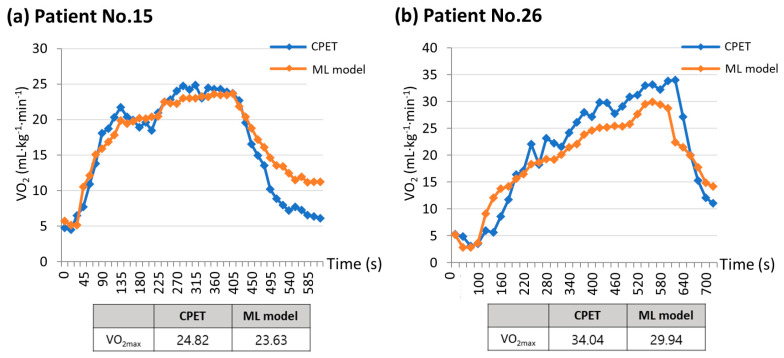
Comparisons between measured VO_2max_ and estimated VO_2max_ using machine learning model. (**a**) Patient No.15 has poor pulmonary function but good exercise function, while (**b**) Patient No.26 has normal pulmonary function and good exercise function. These graphs show that the machine learning model was able to estimate the VO_2max_ value in a manner closely resembling the VO_2_ trend seen in the CPET, regardless of the patient’s initial lung function status. ML model, machine learning model; VO_2max_, maximal oxygen consumption; VO_2_, oxygen consumption; CPET, cardiopulmonary exercise test.

The strength of this study is that it focused on patients scheduled to undergo lung resection, unlike previous studies that primarily targeted healthy young adults. During the CPET, the two patients were compared based on their measured and estimated VO_2max_ values (Figure 6). Figure 6a depicts a patient with poor pulmonary function (FVC, 63% predicted; FEV_1_, 61% predicted) but good exercise function (VO_2max_, 24.82 mL·kg^−1^·min^−1^), while Figure 6b shows a patient with normal pulmonary function (FVC, 98% predicted; FEV_1_, 101% predicted) and good exercise function (VO_2max_, 34.04 mL·kg^−1^·min^−1^). Regardless of the patient’s baseline lung function status, a machine learning model utilizing data collected from a single-lead ECG monitoring device could estimate the VO_2max_ value, with a trend of VO_2_ values very similar to that of the CPET. In assessing the suitability of surgery, relying solely on lung function test results may lead patients with low lung function to opt for an alternative treatment method. However, using the CPET, we determined that the patient could withstand the surgical procedure. When the CPET is not feasible for a patient, a machine learning model that incorporates data from a single-lead ECG patch obtained through an exercise test can provide a VO_2max_ value comparable to that of the CPET.

Although the CPET progressed to stage 7 according to the maximal incremental treadmill protocols, most patients only reached stage 2 or 3. Additionally, some of them failed to establish a substantial plateau at the maximum point of VO_2_, which, to make a strict distinction, signifies VO_2peak_ rather than VO_2max_. However, it should be considered that patients with chronic lung disease and elderly patients were included in this study. Due to their difficulty in maintaining a sufficient plateau, obtaining “the strict” VO_2max_ is very challenging. Furthermore, the maximum heart rate exceeded 80% of the predicted maximum heart rate, indicating that maximal exercise was achieved. From this perspective, we concluded that this can be practically considered as the VO_2max_.

While this study was conducted with a prospective design, it has the limitation of a small sample size of 40. Furthermore, because the study was conducted at a single institution, external validation was not performed. However, this study included patients with a wide range of ages and underlying diseases. Further studies are required for the practical utilization of this device and machine learning models in clinical settings.

## 5. Conclusions

This study aimed to develop a machine learning algorithm to accurately estimate VO_2max_ using a single-lead ECG patch in candidates for lung resection. After exploring multiple combinations, a machine learning model was developed utilizing heart rate, acceleration, and body weight. The VO_2max_ values from the developed model and the FRIEND equation showed lower differences from the reference value. In particular, the developed model, with its strength in more approximate heart rate measurement, provides a closer estimation of VO_2max_ values measured using a CPET regardless of maximal stage level or sex than existing equations. In situations where a CPET is not feasible, this model may be a promising tool for estimating VO_2max_ with greater accuracy and assessing the cardiopulmonary reserve in lung resection candidates.

## Figures and Tables

**Table 1:** The performance of the estimation model using different features.

Number	Feature	R^2^	MAE	MSE	RMSE
5	HR, ACC, AGE, HEIGHT, BW	0.702	2.883	13.698	3.686
4	HR, ACC, AGE, HEIGHT	0.592	3.212	18.644	4.274
	HR, ACC, AGE, BW	0.717	2.770	13.084	3.605
	HR, ACC, HEIGHT, BW	0.662	3.048	15.391	3.888
	HR, AGE, HEIGHT, BW	0.603	3.381	18.735	4.288
3	HR, ACC, AGE	0.688	3.112	17.452	4.131
	HR, ACC, HEIGHT	0.754	2.828	14.103	3.744
	HR, ACC, BW	0.794	2.622	11.773	3.416
	HR, AGE, HEIGHT	0.630	3.424	21.224	4.564
	HR, AGE, BW	0.674	3.189	18.553	4.255
	HR, HEIGHT, BW	0.682	3.259	18.385	4.244
2	HR, ACC	0.770	2.697	13.105	3.607
	HR, AGE	0.678	3.163	18.329	4.224
	HR, HEIGHT	0.717	3.041	16.396	4.038
	HR, BW	0.727	2.960	15.690	3.942
1	HR	0.735	2.882	15.183	3.881

R^2^, R-squared; MAE, mean absolute error; MSE, mean squared error; RMSE, root mean square error; HR, heart rate; ACC, acceleration; BW, body weight.

**Table 2:** The performance of the estimation model using different regression models.

	Linear	Quadratic	Cubic	Ridge	Lasso	Elastic	Random Forest
R^2^	0.670	0.794	0.714	0.670	0.636	0.644	0.679
MAE	3.329	2.622	2.901	3.328	3.443	3.396	3.098
MSE	18.654	11.773	16.383	18.652	20.615	20.121	18.345
RMSE	4.270	3.416	3.925	4.269	4.44	4.396	4.241

R^2^, R-squared; MAE, mean absolute error; MSE, mean squared error; RMSE, root mean square error.

**Table 3:** Baseline characteristics.

Variables	Patients (N = 40)
Age, yr	66.7 ± 8.8
Male	24 (60)
Height, cm	161.4 ± 7.1
Weight, kg	64.4 ± 10.7
Smoking	
Never	21 (52.5)
Ex-smoker	8 (20)
Current smoker	11 (27.5)
Comorbidity	
Coronary artery disease	6 (15)
Heart failure	2 (5)
Arrhythmia	1 (2.5) *
COPD	10 (25)
CCI score	4.6 ± 2.6
PFT	
FVC, L	3.0 ± 0.7
FVC, % predicted	90.7 ± 13.0
FEV_1_, L	2.2 ± 0.6
FEV_1_, % predicted	88.0 ± 18.8
DLco, % predicted	77.7 ± 17.6
6 MWT, m	442.3 ± 57.0
CPET	
Maximal stage	
1	1 (2.5)
2	15 (37.5)
3	22 (55)
4	2 (5)
5–7	0 (0)
Peak heart rate, bpm	133.9 ± 19.0
% predicted maximal HR	87.4 ± 12.4 **
VO_2max_, mL/kg/min	26.3 ± 5.0
ECOG performance status	
0	37 (92.5)
1	3 (7.5)
≥2	0 (0)

Values are expressed as the mean ± standard deviation or number (%); * Atrial fibrillation; ** 80% or more of the predicted maximal HR represents maximal exercise; (References value equations for maximal HR; 220-age); COPD, chronic obstructive pulmonary disease; ILD, interstitial lung disease; CCI, Charlson Comorbidity Index; PFT, pulmonary function test; FVC, forced vital capacity; FEV_1_, forced expiratory volume in 1 s; DLCO, carbon monoxide lung diffusion capacity; 6 MWT, 6-min walking test; CPET, cardiopulmonary exercise test; HR, heart rate; VO_2max_, maximal oxygen consumption; ECOG, Eastern Cooperative Oncology Group.

**Table 4:** Measured and estimated VO_2max_ using machine learning and equations in total and subgroups.

		CPET	ML Model	ACSM	FRIEND	HF-FRIEND
Total (n = 40)	VO_2max_ * [mL·kg^−1^·min^−1^]	26.01 (22.82–30.77)	25.37 (23.49–28.95)	35.77 (24.57–35.77)	29.22 (21.15–29.22)	23.19 (17.39–23.19)
	VO_2max_ Difference ** [mL·kg^−1^·min^−1^]	-	−0.33	5.89	0.30	−5.01
	ICC ***	-	0.693 (0.417–0.838)	0.517 (−0.126–0.780)	0.708 (0.445–0.846)	0.466 (−0.197–0.759)
Maximal Stage 1–2 (n = 14)	VO_2max_ [mL·kg^−1^·min^−1^]	22.99 (19.30–25.16)	23.31 (21.71–24.21)	24.57 (24.57–24.57)	21.15 (21.15–21.15)	17.39 (17.39–17.39)
	VO_2max_ Difference [mL·kg^−1^·min^−1^]	-	0.32	1.24	−2.03	−5.68
	ICC	-	0.499 (−0.677–0.843)	0.301 (−1.072–0.772)	0.229 (−0.858–0.727)	0.081 (−0.242–0.493)
Maximal stage 3–4 (n = 26)	VO_2max_ [mL·kg^−1^·min^−1^]	27.32 (24.93–31.81)	27.84 (25.03–29.82)	35.77 (35.77–35.77)	29.22 (29.22–29.22)	23.19 (23.19–23.19)
	VO_2max_ Difference [mL·kg^−1^·min^−1^]	-	−0.68	8.39	1.56	−4.65
	ICC	-	0.488 (−0.145–0.771)	0.085 (−0.150–0.372)	0.209 (−0.637–0.633)	0.094 (−0.288–0.455)
Male (n = 24)	VO_2max_ [mL·kg^−1^·min^−1^]	26.90 (24.15–31.41)	25.41 (23.73–28.77)	35.77 (24.57–35.77)	29.22 (21.15–2922)	23.19 (17.39–23.19)
	VO_2max_ Difference [mL·kg^−1^·min^−1^]	-	−0.99	6.19	0.25	−5.32
	ICC	-	0.545 (−0.034–0.802)	0.365 (−0.220–0.701)	0.562 (−0.037–0.812)	0.325 (−0.231–0.678)
Female (n = 16)	VO_2max_ [mL·kg^−1^·min^−1^]	23.84 (20.47–26.94)	25.14 (22.50–29.58)	35.48 (24.57–35.77)	28.99 (21.15–29.22)	23.01 (17.39–23.19)
	VO_2max_ Difference [mL·kg^−1^·min^−1^]	-	0.66	5.43	0.38	−4.55
	ICC	-	0.808 (0.453–0.933)	0.660 (−0.176–0.895)	0.818 (0.473–0.937)	0.593 (−0.218–0.867)

* Values are median (Q1–Q3); ** Values are differences between measured and estimated VO_2max_; *** Values are ICC (95% confidence interval); VO_2max_, maximal oxygen consumption, CPET, cardiopulmonary exercise test; ML, machine-learning; ACSM, American College of Sports Medicine; FRIEND, Fitness Registry and the Importance of Exercise National Database; HF, heart failure; Q, quartile; ICC, intraclass correlation coefficient.

## Data Availability

The data presented in this study are available on request from the corresponding author. The data are not publicly available due to a risk of infringement of the patients’ personal information.

## Supplementary Materials

The following supporting information can be downloaded at: https://www.mdpi.com/article/10.3390/healthcare11212863/s1, Figure S1: The process of raw electrocardiogram (ECG) data to measure heart rate. (a) Unstructured ECG raw data are processed through deep-learning semantic segmentation algorithms. (b) The semantic segmentation algorithm accurately differentiates between different ECG waveforms using high-resolution and low-resolution feature maps; Figure S2: (a) Changes in heart rate, acceleration, and gyroscope during stages of CPET. (b) Correlation between VO_2_ and heart rate, acceleration, and gyroscope; Figure S3: Correlation matrix heatmap displaying the Pearson correlation coefficient values for each feature; Table S1: Modified Bruce protocol.
