# Supplementary material for: Development of Machine Learning Model for VO2max Estimation Using a Patch-Type Single-Lead ECG Monitoring Device in Lung Resection Candidates
Source: Healthcare (Basel). 2023 Oct 30;11(21):2863. doi: 10.3390/healthcare11212863 (PMC10648477; doi:10.3390/healthcare11212863)
Supplement: Supplementary file 1 [file healthcare-11-02863-s001.zip › healthcare-2646530-supplementary.pdf]

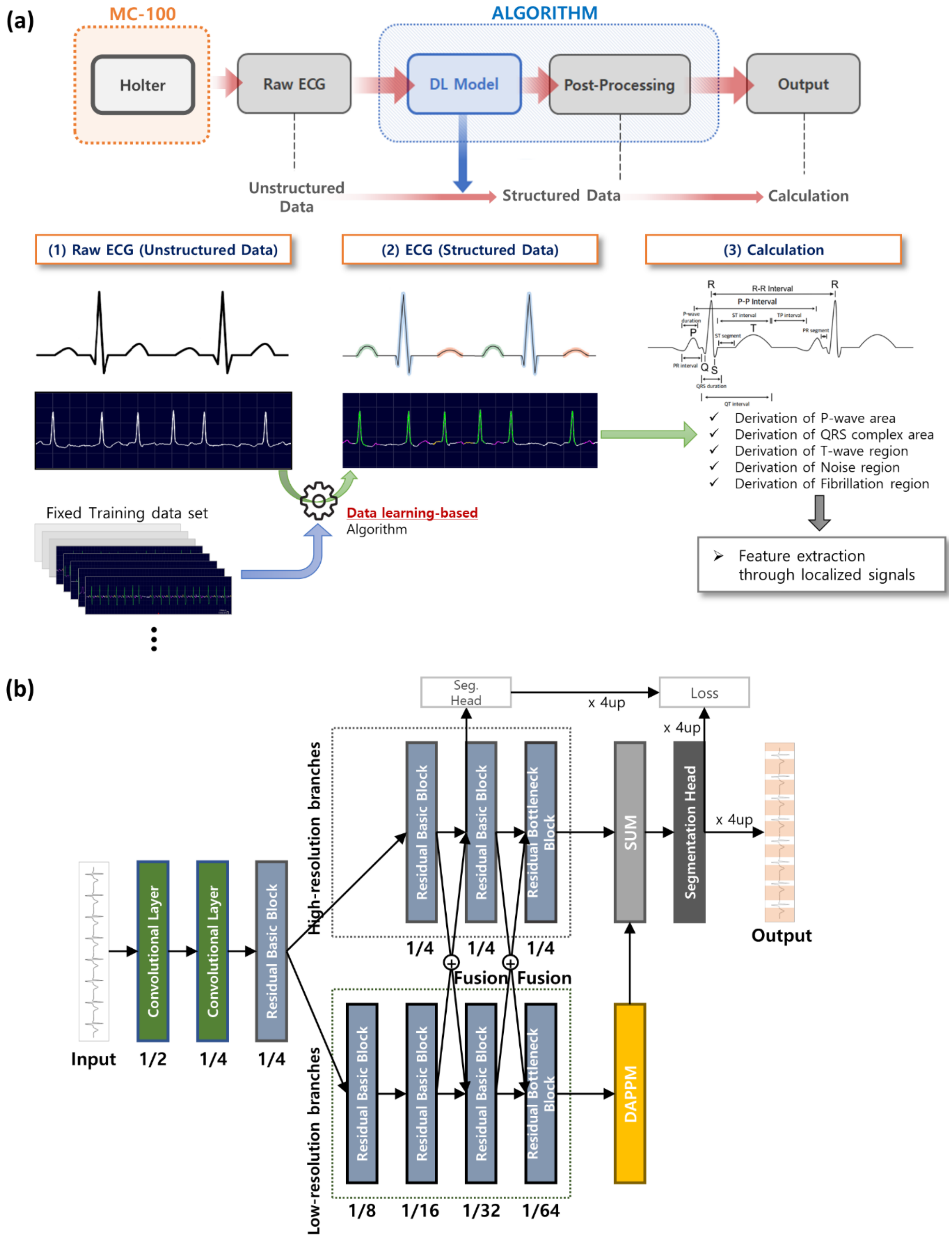

Figure S1. The process of raw electrocardiogram (ECG) data to measure heart rate. (**a**) Unstructured ECG raw data is processed through deep-learning semantic segmentation algorithms. (**b**) The semantic segmentation algorithm accurately differentiates between different ECG waveforms using high-resolution and low-resolution feature maps. This distinction between normal heartbeats and noise allows for the measurement of a heart rate that closely approximates the actual heart rate, even during noisy situations such as exercise. ECG, electrocardiogram; DL Model, deep learning model; Seg. Head, segmentation head; SUM, summation; DAPPM, deep aggregation pyramid pooling module.

(a)

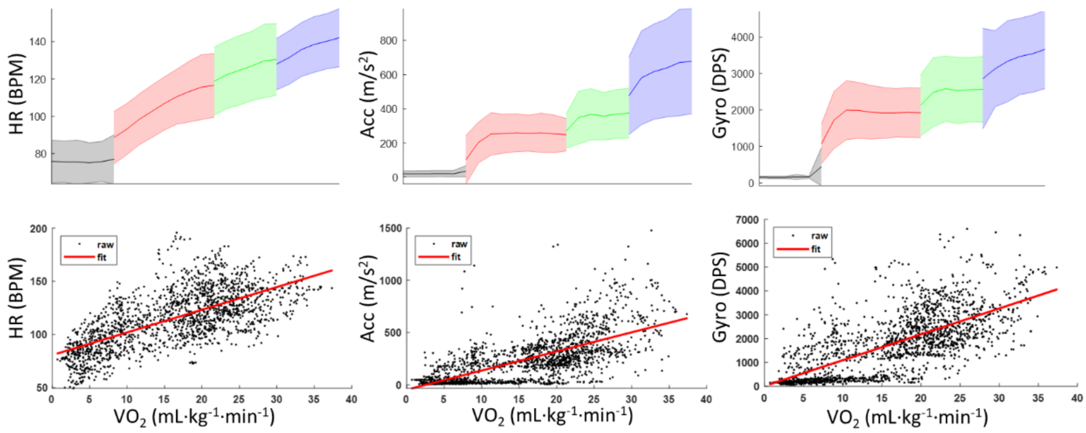

(b)

	VO2 – HR		VO2 – ACC		VO2 – Gyro	
	R	P	R	P	R	P
Pearson	0.83	<0.001	0.70	<0.001	0.87	<0.001

Figure S2. (**a**) Changes in heart rate, acceleration, and gyroscope during stages of CPET. (**b**) Correlation between VO_2 and heart rate, acceleration, and gyroscope. HR, heart rate; ACC, acceleration; GYRO, gyroscope; BPM, beats per minute; DPS, degree per second.

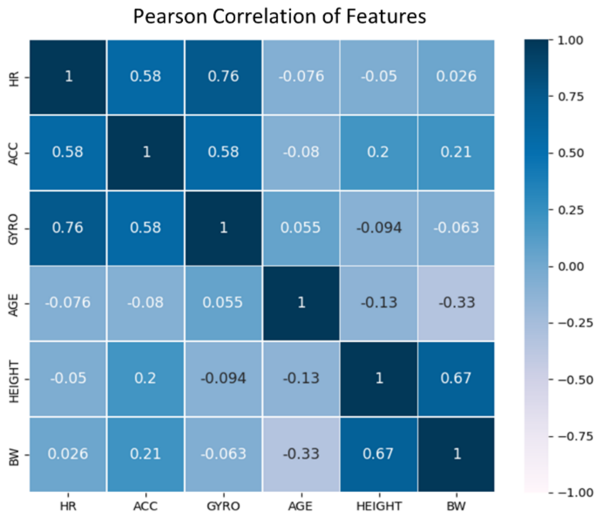

Figure S3. Correlation matrix heatmap displaying the Pearson correlation coefficient values for each feature. As heart rate and Gyro showed high correlation with a Pearson's correlation coefficient of 0.76, Gyro was excluded from feature selection. HR, heart rate; ACC, acceleration; GYRO, gyroscope; BW, body weight.

Table S1. The modified Bruce protocol

Stage	Time (min)	Speed (mph)	Slope (%)
1	0	1.7	10
2	3	2.5	12
3	6	3.4	14
4	9	4.2	16
5	12	5.0	18
6	15	5.5	20
7	18	6.0	22

mph, mile per hour.
